# Inhibition of CD40-TRAF6-dependent inflammatory activity halts the onset of diabetic retinopathy in streptozotocin-diabetic mice

**DOI:** 10.1038/s41387-022-00225-z

**Published:** 2022-10-30

**Authors:** Scott J. Howell, Chieh A. Lee, Thomas E. Zapadka, Sarah I. Lindstrom, Brooklyn E. Taylor, Zakary R. R. Taylor, Katherine G. Barber, Patricia R. Taylor

**Affiliations:** 1 Department of Ophthalmology and Visual Science Case Western Reserve University, School of Medicine, Cleveland, USA; 2 Louis Stokes Cleveland VA Medical Center, Cleveland, OH USA; 3 Present Address: Department of Ophthalmology, Case Western Reserve University, Institute of Pathology, 2085 Adelbert Rd., Room 101, Cleveland, OH USA

**Keywords:** Diabetes complications, Immunology

## Abstract

Diabetes initiates inflammation that can impair the retinal vasculature, and lead to diabetic retinopathy; one of the leading causes of blindness. Inflammatory pathways have been examined as potential therapeutic targets for diabetic retinopathy, but there is still a need for early-stage treatments. We hypothesized that the CD40-TNF Receptor Associated Factor 6 (TRAF6) axis plays a pivotal role in the onset of diabetic retinopathy, and that the CD40-TRAF6 axis would be a prime therapeutic target for early-stage non-proliferative diabetic retinopathy. The CD40-TRAF6 complex can initiate NFκB activation, inflammation, and tissue damage. Further, CD40 and TRAF6 are constitutively expressed on Muller glia, and upregulated in the diabetic retina. Yet the role of the CD40-TRAF6 complex in the onset of diabetic retinopathy is still unclear. In the current study, we examined the CD40-TRAF6 axis in diabetic retinopathy using a small molecule inhibitor (SMI-6877002) on streptozotocin-induced diabetic mice. When CD40-TRAF6-dependent inflammation was inhibited, retinal vascular leakage and capillary degeneration was ameliorated in diabetic mice. Collectively, these data suggest that the CD40-TRAF6 axis plays a pivotal role in the onset of diabetic retinopathy, and could be a novel therapeutic target for early diabetic retinopathy.

## Introduction

Inflammation can impair the retinal microvasculature, and lead to diabetic retinopathy [1]. IL-17A is an inflammatory protein that plays a role in the onset of diabetic retinopathy [2]. Normally, IL-17A is not constitutive [3]. However, it is continuously produced in diabetics [4]. Recently, we reported that IL-17A enhances retinal vascular leakage and capillary degeneration in diabetic mice [2, 5].

When IL-17A binds to its receptor, it can initiate TNF Receptor Associated Factor 6 (TRAF6) to bind to CD40. The CD40-TRAF6 complex then initiates NFκB activation, inflammation, and tissue damage [6]. Both CD40 and TRAF6 are constitutively expressed in the retina, and upregulated during diabetes [7, 8]. Thus, we hypothesized that the CD40-TRAF6 axis would be an optimal therapeutic target for early-stage diabetic retinopathy.

We investigated the CD40-TRAF6 axis in the onset of diabetic retinopathy in streptozotocin (STZ)-induced diabetic mice using a small molecule inhibitor (SMI-6877002), which can pass the blood-retina barrier [9, 10]. SMI-6877002 causes a conformational change in the binding groove of TRAF6 upon CD40 binding, which inhibits the CD40-TRAF6 complex from initiating NFκB activation [9]. This causes a decrease in inflammation and suppresses TNF-α, which is produced in early-stage retinal pathogenesis and a precursor to diabetic retinopathy [9–11]. A weekly subcutaneous injection of 100 μl of saline containing 20 μM of SMI-6877002 was sufficient to halt TNF-α and VEGF production in Muller glia and the retina. When SMI-6877002 was administered throughout an 8-month duration in STZ-diabetic mice, retinal vascular leakage and capillary degeneration was halted. These findings suggest that the CD40-TRAF6 axis could be a novel therapeutic target for early-stage diabetic retinopathy.

## Materials & methods

### Diabetic mice

Streptozotocin (60 mg/kg) was injected on five consectutive days in 8–10 week-old C57BL/6 mice as previously described [12, 13]. Diabetes was confirmed by blood glucose higher than 250 mg/dl and hemoglobin A1C levels. SMI-6877002 treatment was administered after streptozotocin damaged the pancreatic beta cells, which allows diabetic retinopathy to develop. CWRU IACUC approved animal protocols with a power calculation = 0.9, which have been strictly followed.

### SMI-6877002 treatment

**Fig. 1:**
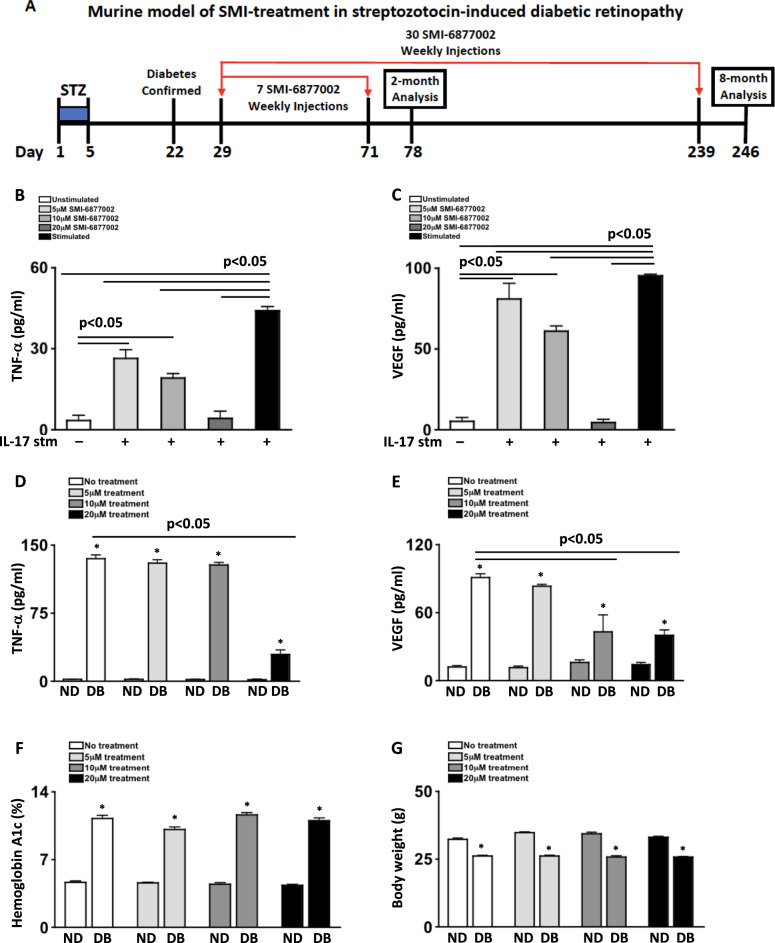
Inflammation in Muller glia and diabetic mice receiving SMI-6877002 treatments. **A** Schematic of diabetic retinopathy murine model and SMI-6877002 treatment regimen. Quantifications of TNF-α (**B**) and VEGF (**C**) in supernatants of unstimulated (white), or IL-17A stimulated human Muller glia that were untreated (black), received 5 μM (light grey), 10 μM (mid-grey), or 20 μM (dark grey) of SMI-6877002 (*n* = 6/group). Levels of TNF-α (**D**) and VEGF (**E**) in retinas (*n* = 3/group), and hemoglobin A1c (**F**) and body weight (**G**) of non-diabetic (ND) and STZ-diabetic (DB) mice that received no treatment (white), 5 μM (light grey), 10 μM (dark grey), or 20 μM (black) of SMI-6877002 injections 1 time a week; 2-months after diabetes was confirmed. Error bars represent the SEM, * = *p* < 0.01; all p-values were equated using two-way ANOVA and unpaired student’s t-test. Data are representative of 2 separate experiments.

SMI-6877002 (3-((2,5-Dimethylphenyl) amino)-1-phenyl-2-propen-1-one, (2E)-3-((2,5-Dimethylphenyl) amino)-1-phenyl-2-propen-1-one) is a cell-permeable propenone that causes a conformational change in the Arg466 residue, altering the binding groove of the CD40-TRAF6 complex. This halts NFκB activation and reduces inflammation [9]. Levels of TNF-α are significantly decreased when SMI-6877002 is properly administered [9, 10]. To establish the proper treatment regimen of SMI-6877002 (Fig. 1A), 100 μl of saline containing 5, 10, or 20 μM of SMI-6877002 was subcutaneously injected once weekly in STZ-diabetic mice, and levels of TNF-α were quantified in pg/ml by ELISA per manufacturer’s instructions (R&D). SMI-6877002 toxicity was defined by lethargy, body weight, respiratory stress, autopsy organ appearance, and mortality rate.

### SMI-6877002 treatment of Muller glia

Human Muller glia were obtained from human cadavers (Eversight). The posterior section of the retinal globes were mechanically disrupted and incubated in DMEM/HAM F12 media at 37 C with 5% CO_2_ for 2 weeks. Cell purity of > 95% GLAST^+^/Vimentin^+^ Muller glia was confirmed by flow cytometry. Muller glia were incubated with SMI-6877002 2 h prior to a 100 ng/ml stimulation of recombinant IL-17A for 18 h. Supernatants were collected for TNF-α and VEGF ELISA analysis.

### Retinal vascular leakage

Retinal vascular leakage was determined as previously described [2, 12]. FITC-BSA (100 μg/gram body weight) was intravenously injected, circulated for 20 min, and retinas (n *=* 7/group) collected, fixed, and mounted in OCT. Sections were analyzed via fluorescent microscopy and flourescent intesity was determined using Metamorph Imaging Software (Molecular Devices). Plasma levels of FITC-BSA were used to nomalize fluorescence between individual animals.

### Capillary degeneration in retina

Acellular capillaries were counted in five fields per retina, as previously described [12–15]. Enucleated eyes were fixed, digested in elastase for 2 h, and immersed in Tris buffer (pH=8.5) for 16 h at 37 C. Capillary beds were extracted by mechanical disruption, stained, (hematoxylin and periodic acid-Schiff) and viewed at 200x magnification on brightfield microscope.

### Pericyte quantification

Pericyte ghosts were counted in the above-mentioned capillary beds. Alternatively, two pooled retinas per mouse (*n* = 3/group) were digested in papain (Worthington) and then collaganase (80 U/ml; Sigma Aldrich) to collect cells. Cells were stained with PE-conjugated, anti-mouse PDGFRβ (Abcam) antibody for flow cytometry analysis (C6 Accuri flow cytometer). Gates were set to an isotype control, and PDGFRβ^+^ pericytes were quantified.

### Statistical analysis

Prism software (Graph pad) was used to conduct two-way ANOVA and unpaired t-test with Tukey’s post-hoc analysis, p-values < 0.05 are marked as significant.

## Results and discussion

Muller glia constitutively express CD40, TRAF6, and the IL-17 receptor [2, 7, 8]. Also, IL-17A can induce Muller glia to produce TNF-α and VEGF [2]. To detemine the proper SMI-6877002 treatment regimen, levels of TNF-α and VEGF in the spent media of IL-17A-stimulated human Muller glia with or without SMI-6877002 treatment were quantified by ELISA. Unstimulated Muller glia produced negligible levels of TNF-α (Fig. 1B). However, Muller glia stimulated with IL-17A produced ~50 pg/ml of TNF-α, which was significantly decreased to ~30 pg/ml and ~20 pg/ml when treated with 5 μM and 10 μM of SMI-6877002 respectively. Muller glia treated with 20 μM of SMI-6877002 halted TNF-α production. Additionally, negligible levels of VEGF was detected in the supernatants of unstimulated Muller glia. Yet, Muller glia produced ~100 pg/ml of VEGF when stimulated with IL-17A. This was significantly decreased to ~80 pg/ml and ~60 pg/ml when the cells were treated with 5 μM and 10 μM of SMI-6877002, respectively, and halted in 20 μM treated Muller glia (Fig. 1C). The ablation of TNF-α and VEGF production affirms that inflammatory activity of the CD40-TRAF6 complex is halted when 20 μM of SMI-6877002 is administered.

To determine the in vivo SMI-6877002 treatment regimen, weekly subcutaneous injections of 100 μl of saline containing 5 μM, 10 μM, or 20 μM of SMI-6877002 were administered to non-diabetic and STZ-diabetic mice after diabetes was confirmed (Fig. 1A). Levels of TNF-α and VEGF in retinal protein lysates were anlayzed by ELISA; 2-months post-diabetes. Negligible levels of TNF-α was detected in the retina of all non-diabetic mice, while ~140 pg/ml of TNF-α was detected in the retina of untreated, 5 μM, and 10 μM SMI-6877002 treated diabetic mice. Levels of TNF-α were significantly decreased to ~30 pg/ml in the retina of 20 μM SMI-6877002 treated diabetic mice (Fig. 1D). Also, ~20 pg/ml of VEGF was detected in the retina of all non-diabetic mice. While the level of VEGF in the retinas of untreated and 5 μM SMI-6877002 treated diabetic mice was significantly higher at ~80 and ~75 pg/ml, respectively. VEGF was significantly decreased in the retina of 10 μM and 20 μM SMI-6877002 treated diabetic mice in a dose-dependent manner to ~55 pg/ml and ~40 pg/ml, repsectively (Fig. 1E). This suggests that 20 μM of SMI-6877002 is sufficient to decrease inflammatory precursors to diabetic retinopathy [11, 16].

**Fig. 2:**
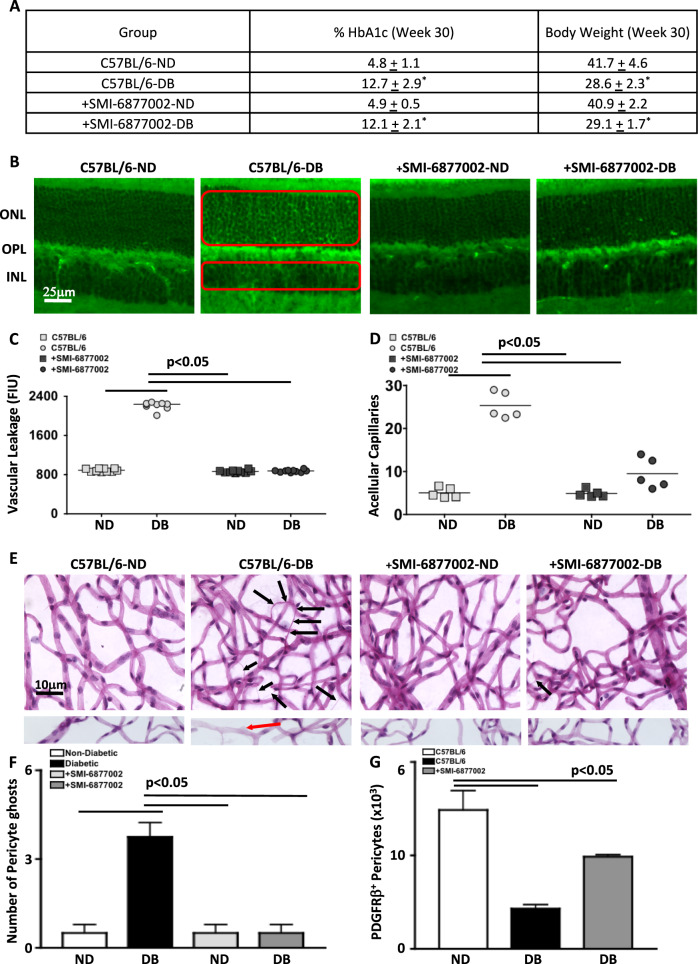
Vascular leakage, capillary degeneration, and pericyte death in the retinas of diabetic mice. **A** Clinical data of hemoglobin A1c and body weight of mice 30-weeks after diabetes was confirmed. Treated mice received 30 injections of 20 μM SMI-6877002. **B** Representative fluorescent microscopy of vascular leakage (highlighted by two red boxes in leakage areas) in retinal cross-sections of outer nuclear layer (ONL), outer plexiform layer (OPL), and inner nuclear layer (INL) in untreated and treated non-diabetic and diabetic mice. Quantification of vascular leakage (**C**), and acellular capillaries (**D**) in each retina of untreated non-diabetic (light grey squares), untreated diabetic (light grey circles), treated non-diabetic (black squares), and treated diabetic (black circles) mice. Scale bars of images = 25 μm. Each data point represents an individual retina from 7 different mice; 8-months post-diabetes. **E** Representative images of acellular capillaries (upper panel; 5 acellular capillaries in C57BL/6 DB and 1 acellular capillary in SMI-6877002 treated DB mice are highlighted), and pericyte ghosts (lower panel) in the retinal capillary beds of untreated and treated non-diabetic and diabetic mice. Black arrows highlight acellular capillaries (upper panel) and red arrows highlight pericyte ghosts (lower panel). Scale bars of images = 10 μm. **F** Quantification of pericyte ghosts in retinal capillary beds (*n* = 5/group). **G** Quantification of PDGFRβ^+^ pericytes in total retina per flow cytometry analysis (*n* = 3/group). Error bars represent the SEM, and *p*-values were equated by two-way ANOVA analysis and unpaired t-test with Tukey’s post-hoc analysis. Data are representative of 2 experiments.

Seventeen days after the final STZ-injection, hemoglobin A1c levels were examined. All diabetic mice had significantly higher A1c levels than non-diabetic mice (*n* = 9/group). But there were no differences between SMI-6877002 treated diabetic mice and untreated diabetic mice (Fig. 1F). A healthy body weight in the STZ-diabetic mice is maintained, yet it is normally lower than the non-diabetic controls [12, 17]. As shown in Fig. 1G, all STZ-diabetic mice displayed a significantly lower body weight than all non-diabetic mice. However, there were no differences in the body weight of SMI-6877002 treated and untreated diabetic mice. Nor was there any other toxicity observed in any of the SMI-6877002 treated mice. Similarily, all diabetic mice had significantly higher hemoglobin A1c levels and significantly lower body weight than the nondiabetic controls (Fig. 2A), and there were no differences in the untreated versus treated diabetic mice; 30 weeks post-diabetes. Collectively, these observations confirm that a weekly treatment of 20 μM of SMI-6877002 is sufficient to halt CD40-TRAF6-depenedent inflammation, without impacting diabetic conditions or eliciting toxicity in this murine diabetes model.

Vascular leakage is one of the earliest clinical symptoms of diabetic retinopathy detected in diabetics, and in this murine model 8-months post-diabetes [17–20]. Vascular leakage is indicated by diffuse hyper-fluorescence in the outer nuclear layer (ONL), outer plexiform layer (OPL), and the inner nuclear layer (INL) of the retina, as highlighted with two red boxes in leakage areas (Fig. 2B**)**. Quantification of vascular leakage, using fluorescent intensity units (FIU), was significantly increased in all retina layers of the untreated diabetic mice than all non-diabetic mice. While retinal vascular leakage was significantly decreased in the SMI-6877002 treated diabetic mice to similar levels of non-diabetic mice (Fig. 2C). This indicates that the CD40-TRAF6 complex plays a pivotal role in retinal vascular leakage.

In non-proliferative diabetic retinopathy and in this 8-month STZ-diabetes model, retinal capillaries degenerate and vascular cells die [12, 20]. Capillary degeneration was examined in the retinal capillary beds (*n* = 7/group) of untreated and SMI-treated non-diabetic and diabetic mice, by counting the number of acellular capillaries (highlighted by black arrows in the upper panel of Fig. 2E). The number of acellular capillaries in the retinas of untreated diabetic mice was significantly higher than the number in all nondiabetic and diabetic SMI-treated mice (Fig. 2D). Further, there was no significant difference in acellular capillaries between the SMI-6877002 treated diabetic mice and any of the non-diabetic mice (Fig. 2D). Additionally, the number of pericyte ghosts in these capillary beds were quantified (highlighted by red arrow in the lower panel of Fig. 2E). There was a significantly higher number of pericyte ghosts in the capillaries of untreated diabetic mice than all non-diabetic mice and the SMI-6877002 treated diabetic mice (Fig. 2F). Conversely, the number of viable PDGFRβ^+^ pericytes were quantified in the retinas (*n* = 3 samples/group) of untreated nondiabetic (white), untreated diabetic (black), and SMI-6877002 treated (dark grey) mice by flow cytometry. The number of pericytes in the retinas of nondiabetic mice was significantly higher than the number of pericytes in the retinas of all diabetic mice (Fig. 2G). However, there was a significantly higher number of pericytes in the SMI-6877002 treated diabetic mice than the untreated diabetic mice. Suggesting that the CD40-TRAF6 axis plays a role in hyperglycemic-driven pericyte death. Collectively, all of the results provide evidence that the CD40-TRAF6 axis plays a pathologic role in retinal inflammation, vasoregression, and pericyte death, which is representative of the onset of early-stage diabetic retinopathy. Further suggesting that the CD40-TRAF6 axis could be a good therapeutic target for early-stage diabetic retinopathy.

## Data Availability

The datasets generated during and/or analyzed during the current study are available from the corresponding author on reasonable request.
